# Comparative Characterization of Cardiac Development Specific microRNAs: Fetal Regulators for Future

**DOI:** 10.1371/journal.pone.0139359

**Published:** 2015-10-14

**Authors:** Yashika Rustagi, Hitesh K. Jaiswal, Kamal Rawal, Gopal C. Kundu, Vibha Rani

**Affiliations:** 1 Department of Biotechnology, Jaypee Institute of Information Technology, A–10, Sector–62, Noida, 201307, Uttar Pradesh, India; 2 Laboratory of Tumor Biology, Angiogenesis and Nanomedicine Research, National Centre for Cell Science (NCCS), Pune 411007, India; Kunming University of Science and Technology, CHINA

## Abstract

MicroRNAs (miRNAs) are small, conserved RNAs known to regulate several biological processes by influencing gene expression in eukaryotes. The implication of miRNAs as another player of regulatory layers during heart development and diseases has recently been explored. However, there is no study which elucidates the profiling of miRNAs during development of heart till date. Very limited miRNAs have been reported to date in cardiac context. In addition, integration of large scale experimental data with computational and comparative approaches remains an unsolved challenge.The present study was designed to identify the microRNAs implicated in heart development using next generation sequencing, bioinformatics and experimental approaches. We sequenced six small RNA libraries prepared from different developmental stages of the heart using chicken as a model system to produce millions of short sequence reads. We detected 353 known and 703 novel miRNAs involved in heart development. Out of total 1056 microRNAs identified, 32.7% of total dataset of known microRNAs displayed differential expression whereas seven well studied microRNAs namely let–7, miR–140, miR–181, miR–30, miR–205, miR–103 and miR–22 were found to be conserved throughout the heart development. The 3’UTR sequences of genes were screened from *Gallus gallus* genome for potential microRNA targets. The target mRNAs were appeared to be enriched with genes related to cell cycle, apoptosis, signaling pathways, extracellular remodeling, metabolism, chromatin remodeling and transcriptional regulators. Our study presents the first comprehensive overview of microRNA profiling during heart development and prediction of possible cardiac specific targets and has a big potential in future to develop microRNA based therapeutics against cardiac pathologies where fetal gene re-expression is witnessed in adult heart.

## Introduction

The gene expression program during embryonic development is highly orchestrated process that entails precise control of large number of gene network [1]. Heart development, a highly conserved process from flies to human is the first sign of organogenesis in the developing embryos which leads to functional circulatory system essential for survival of organisms [2–3]. It is a tightly regulated complex process requiring exquisite control of transcriptional programs [4]. During the years, heart development has come up as a paradigm of cell differentiation and organogenesis and chicken embryos have emerged as classical model for understanding the vertebral organogenesis and development due to characteristically similar organ development [5]. In addition, developmental processes of chicken embryos are well defined and live developing chick embryos can be easily manipulated in ovum [6–7].

The precise spatio-temporal regulation of gene expression was earlier believed to be regulated by a diverse set of transcription factors, chromatin regulators, and signalling molecules [8–9]. Recently, microRNAs, a class of non-coding RNA molecules, have drawn considerable attention for their prominent role in development and diseases by regulating the expression of target mRNAs [10–15]. The discovery of microRNAs and their regulated function in various biological events have therefore revealed new insight in to the complexities involved in the gene regulatory network during development [16–17]. MicroRNAs regulate expression of genes through sequence specific targeting of the 3’ untranslated regions of target mRNAs by either inhibiting translation or inducing mRNA degradation resulting in translational repression and gene silencing [18]. The current reports indicate the existence of hundreds of microRNA genes in the vertebrate genome which regulate approximately 30% of mRNAs [19]. Therefore, identification of comprehensive sets of miRNA and their targets during the organ development is a critical step to facilitate our understanding of genome organisation, cell growth, differentiation, organ development and diseases [20–21].

The present study was designed to understand the mechanisms of heart development and its reprogramming and to identify the microRNAs implicated in heart development. The study hold a big potential in future to develop microRNA based therapeutics against cardiac pathologies, as fetal re-expression of cardiac genes is witnessed in number of cardiac diseases, leading causes of morbidity and mortality worldwide [22–24].

No such developing heart specific comparative study has been conducted in the past. Previous studies were conducted to identify the small regulatory RNAs expressed in the whole embryos collected at day 5, 7 and 9 of incubation [25]. Another study was conducted by Hick et al in 2008, where a small RNA library from 11-day old chick embryos was constructed to examine the miRNA expression profiles of the whole embryos [26]. Darnell et al in 2006, employed high-throughput whole mount in situ hybridization on 0.5 to 5 days old chicken embryos to map expression of 135 miRNA genes [27]. In all such studies, non-specific microRNAs pool, regulating the development of whole organism was used and hence specificity with respect to particular organ development is lacking. In the present study we aimed to characterize stage specific microRNAs regulating the heart development. We identified several novel cardiac specific miRNAs during various stages of heart development using next generation sequencing technology. We also aimed to identify the time when miRNA population became involved in heart development using differential expression. Through this analysis, we present a functional categorization of mRNA targets of screened cardiac miRNAs. We also show the enrichment of genes as well as pathways of muscle development and apoptosis in the data-sets. Importantly, this is the first study conducted on developing heart rather than using whole embryo, that would not only increase the efficacy and sensitivity of regulation of fetal cardiac gene program, but would also enrich the learning and findings related to cardiac specific microRNAs and their mRNA targets.

## Material & Methods

### Source of Chemicals

Chemicals were purchased from Sigma-Aldrich,USA unless or otherwise stated.

### RNA isolation from chicken hearts

Fertilized *Gallus gallus*chicken eggs were obtained from Hatchery, Faridabad, Haryana, India. The eggs were incubated in incubator at 37.5°C with more than 90% humidity and with rotations every 6 h. Chick embryos were collected at four, six, eight, ten, twelve and fourteenth days of incubation [CHL1–CHL6]. These represent the chick embryonic developmental stages HH24, HH29, HH34, HH36, HH38 and HH40 respectively, which cover various stages of cardiogenesis [28–29]. Eggs were placed in a hood by maintaining its horizontal position in the glass dish and wiped with 70% ethanol. Egg shells were punctured with blunt ended curved forceps using sterile conditions. 1–2 ml of white albumen was withdrawn, which allowed the embryos to move away from the upper surface of the eggs and further carefully opened the taped-covered top of the eggs with the tip of scissor. After the removal of the egg shell, embryos were rinsed in cold and sterilized 1 XPBS, vitelline membranes were taken off from the embryos and yolks were cleaned. After removal of extra embryonic membranes, developing hearts from various stages were carefully separated from embryo without removing any other tissues using micro dissecting scissors and immediately processed for total RNA isolation using TRIzol Reagent(Sigma Aldrich, USA) after crushing the heart using sterile glass homogenizers. Integrity of extracted stage specific RNA pools were checked on 1X MOPS-formaldehyde agarose gel and quality was verified by Agilent 2000 bioanalyzer. Low molecular weight RNAs were extracted using mirVana small RNA isolation kit (Ambion, Life technologies, USA) as per instructions. RNAs concentrations and purities were determined spectrophotometically by measuring A260/A280 ratio using the NanoDrop ND–1000 spectrophotometer (Nanodrop Technologies, USA). The small RNA fractions were confirmed by using Urea-PAGE gel. RNA samples were stored at –80°C until further use.

### Small RNA library construction and high throughput sequencing

Total six cardiac heart libraries (CHL1-CHL6) were prepared for deep sequencing and subsequent characterization (S1 Fig). For each heart developmental stage, equal quantities [20μg] of small RNAs were submitted to Illumina Inc. for small RNA deep sequencing. In brief, the low-molecular- weight RNA was precipitated with PEG8000/NaCl. RNA was purified by polyacrylamide gel electrophoresis [PAGE] to elute molecules in the range of 18–30nt. The extracted RNAs were ligated to the 3′ adopter [TCGTATGCCGTCTTCTGCTTG]. The ligated RNAs from each library were eluted from 15% denatured polyacrylamide gel using 0.3NNaCl, followed by the ligation of 5′ adopter [GTTCAGAGTTCTACAGTCCGACGATC]. The samples were further used as templates for cDNA synthesis using SuperScriptH III reverse transcriptase (Illumina Inc., USA) by using primers complementary to 5’ and 3’ linker sequences with 25 PCR cycles of 95°C for 30s, 50°C for 30s, and 72°C for 30s. The PCR products were extracted from the agarose gels using the Gel Extraction Kit (Qiagen Ltd., UK) and sequenced by Illumina sequencing technology after purification.

### Sequence analysis

Illumina platform generates library sequence reads with the base quality scores [30]. The datasets were filtered to remove redundant sequences. A filtered set of the unique reads along with read counts were labelled as sequence tags. We used UCSC genome browser for *Gallus gallus* v.2.1 genome sequence and annotations [31]. Sequence tags were mapped onto chicken genome assembly using BLAT software after trimming the adaptors sequences. Only tags with perfect match or with one mismatch were considered for further analysis. The tags were mapped to chicken genome particularly in context to important genomic features such as exons, introns, repeats, rRNAs, tRNAs, snRNAs, and snoRNAs [32–34]. All the library sequences were initially normalized by count-per-million method using DEseq tool where library sequences were adjusted for differences in sequencing depth. The counts were then normalized to reads per million to facilitate comparison between libraries.

### MicroRNA analysis

miRBase version 19.0 was searched for identification of small RNA library sequences with insert length 18 to 28 bases [http://microrna.sanger.ac.uk] followed by filtration. Next, the filtered sets were compared with miRNAs and miRNA precursor sequences of all organisms [35]. We classified our miRNAs datasets into conserved/differentially expressed known and novel cardiac specific microRNAs. The representative microRNAs were aligned to *Gallus gallus* genome as well as transcriptome to identify their precursors. RNA fold server using MEF RNA structure prediction algorithm was used to identify hairpin structures of predicted microRNAs to identifying bonafide microRNAs from spurious sequences [36]. Comprehensive information for each microRNA was collected with respect to its location, strand, sequence, chromosome, miRNA* [the sequences originating from the RNA hairpin arm opposite to the annotated mature miRNA containing arm], hairpin sequence, abundance, sRNA length, hairpin length, hairpin structure, minimum free energy, G/C %, miRBase ID and P_Val. Expression profiles for conserved, differential and novel microRNAs isolated from all six libraries were generated from a comprehensive analysis using DEGseq and heat maps were generated using “R”[37]. DEGseq is a free R package for identifying differentially expressed miRNAs from RNA-seq data. The method DEGseq took uniquely mapped reads from RNA-seq data of six libraries with whole chicken sequenced dataset as input. The text file asoutput of DEGseq contained the miRNAs expression values for all the library samples with a P-value.

### Expression analysis by Real Time PCR

Expression levels of the seven conserved mature miRNAs were determined by quantitative real time PCR(Piko-Real Time 96 Thermofischer Scientific, Waltham, MA USA) using Taqman microRNA Reverse transcription kit and SYBR Green qPCR Master Mixes (Thermo Scientific) with the designed primers listed. (S1 Table). The small nuclear RNA U6B was used as an internal control (RNU6B). The reactions were performed in a 96-well optical plate in two cyclic program at 95°C for 30 sec, followed by 40 cycles of 95°C for 15 sec and 55°C for 30 sec. All reactions were run in triplicates. After reaction, the threshold cycle (Ct) was determined by using the comparative Ct method where the amount of targeted miRNAs was normalized to the endogenous control sample by 2-ΔΔCt. The Ct was defined as the fractional cycle number at which the fluorescence passed a fixed threshold.

### Chromosomal distribution of miRNAs

MiRNAs were used to map on to whole chicken genome [http://genome.ucsc.edu/cgi-bin/hgGateway?org=chicken] and graphs were created implementing number of miRNAs mapped on a chromosome.

### Computational prediction of miRNA targets

We used standard tools such as target scan to predict targets of miRNAs with following conditions *(i)* Perfect match in the seed region *(ii)* 8nt from the 5’ end of miRNA *(iii)* The minimum free energy of the miRNA/ target duplex was less than -20 Kcal/mol*(iv)* The total score for a miRNA-mRNA pairs was greater than 100 [38]. We also used PicTar as an additional tool for validation of predicted targets that works on Hidden Markov model and uses alignment to eight vertebrate species to eliminate false positives [39–40].

### GO Analysis of the predicted miRNA target genes

To enable the functional analysis of the potential targets of identified miRNAs, we used BLAST2GO tool. We collected the common predicted miRNA targets in all the cardiac specific libraries. Given a set of genes, the tool extracted GO terms to classify them as biological processes, molecular functions and cellular processes as keywords that were over represented in the set of the genes. The BLAST2GOgene annotation tool generated results in terms of predicted GO cluster, GO term, number of targets, gene description, enrichment fold and P_value [41–44].

### KEGG Pathway analysis of the predicted miRNA target genes

To understand miRNAs and their target genes at biological network level, we used the KEGG [Kyoto Encyclopedia of Genes and Genomes] tool called KAAS [KEGG Automatic Annotation Server] to automate the processes of the K number assignment and the subsequent pathway mapping. Query genes [target mRNAs] were searched against the reference datasets. Candidate hits were selected based upon BLAST scores and the bi-directional hit rate. These hits were divided into KO groups and query sequences were assigned to these KO groups based upon standard scoring system [45].

### Statistical analysis

The results were presented as mean ± standard error of the mean (s.e.m). Microsoft Excel SPSS and PRISM software was used to perform all statistical analysis. The R software was used for all statistical computing and graphics. For the expression analysis, reads data were log10 and log2-transformed and for comparison of six experimental groups, ANOVA-test was used. Adjustment of the p-value by multiple comparisons was performed by calculating false discovery rates (FDR). Those miRNAs with FDR<0.1 were extracted as differentially expressed. Hierarchical clustering was performed using an R platform and heat mapswere generated.

### Ethics Statement

All animal work was performed in accordance with local ethical guidelines. The work was conducted on developing chick embryos. Chick embryos are increasingly recognised as model of choice for mammalian biology as no ethical approval is required for working with developing chick embryos up to day 15 since they lack immune system in early developmental stages and do not experience pain as per NIH guidelines for avian embryos use.

## Results

### High-throughput sequencing of small RNAs from developing chick hearts

**Fig 1:**
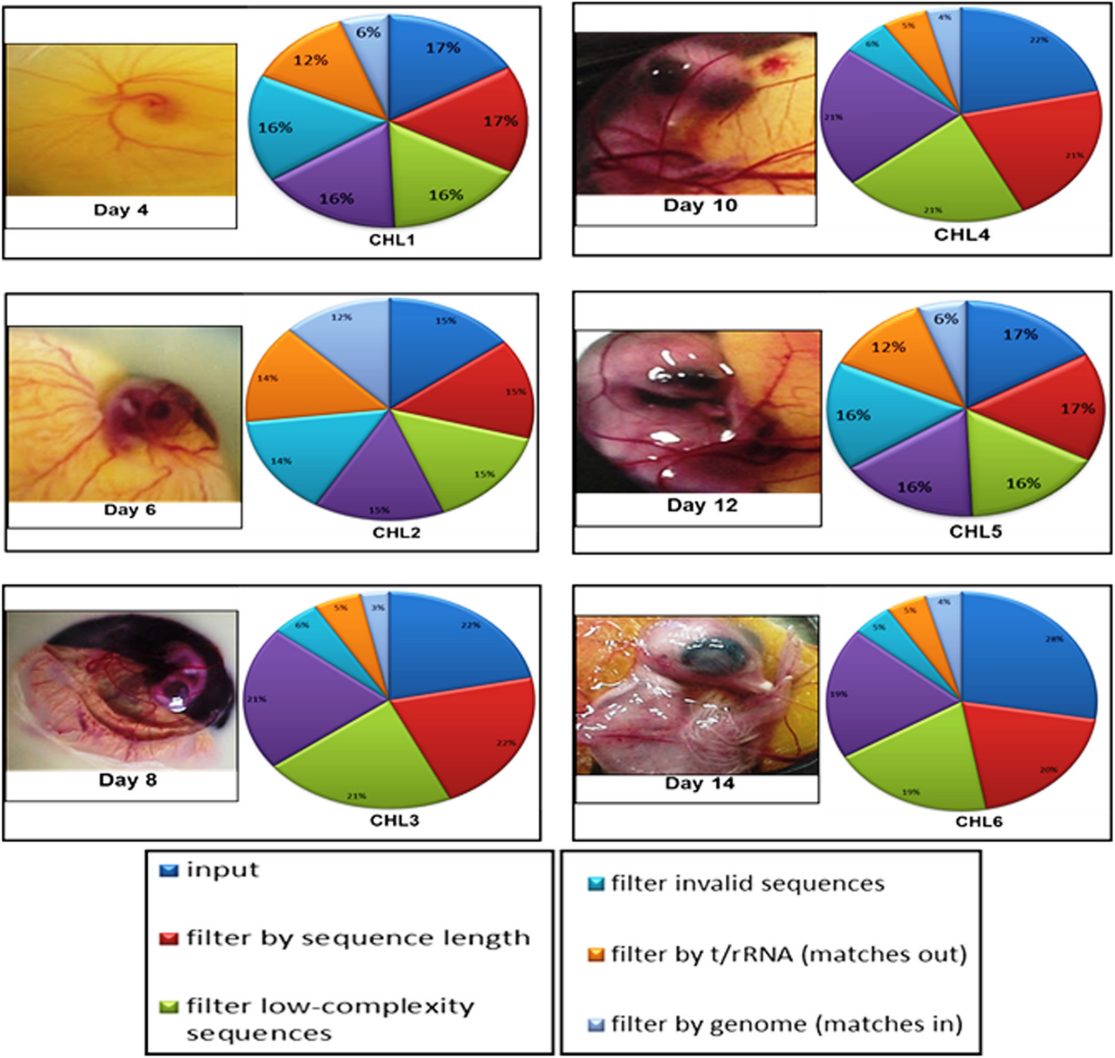
Transcript filters analysis of six heart libraries. All reads from six libraries produced from deep sequencing were aligned to the chicken genome to find the genuine reads and filtered for analysis.

**Fig 2:**
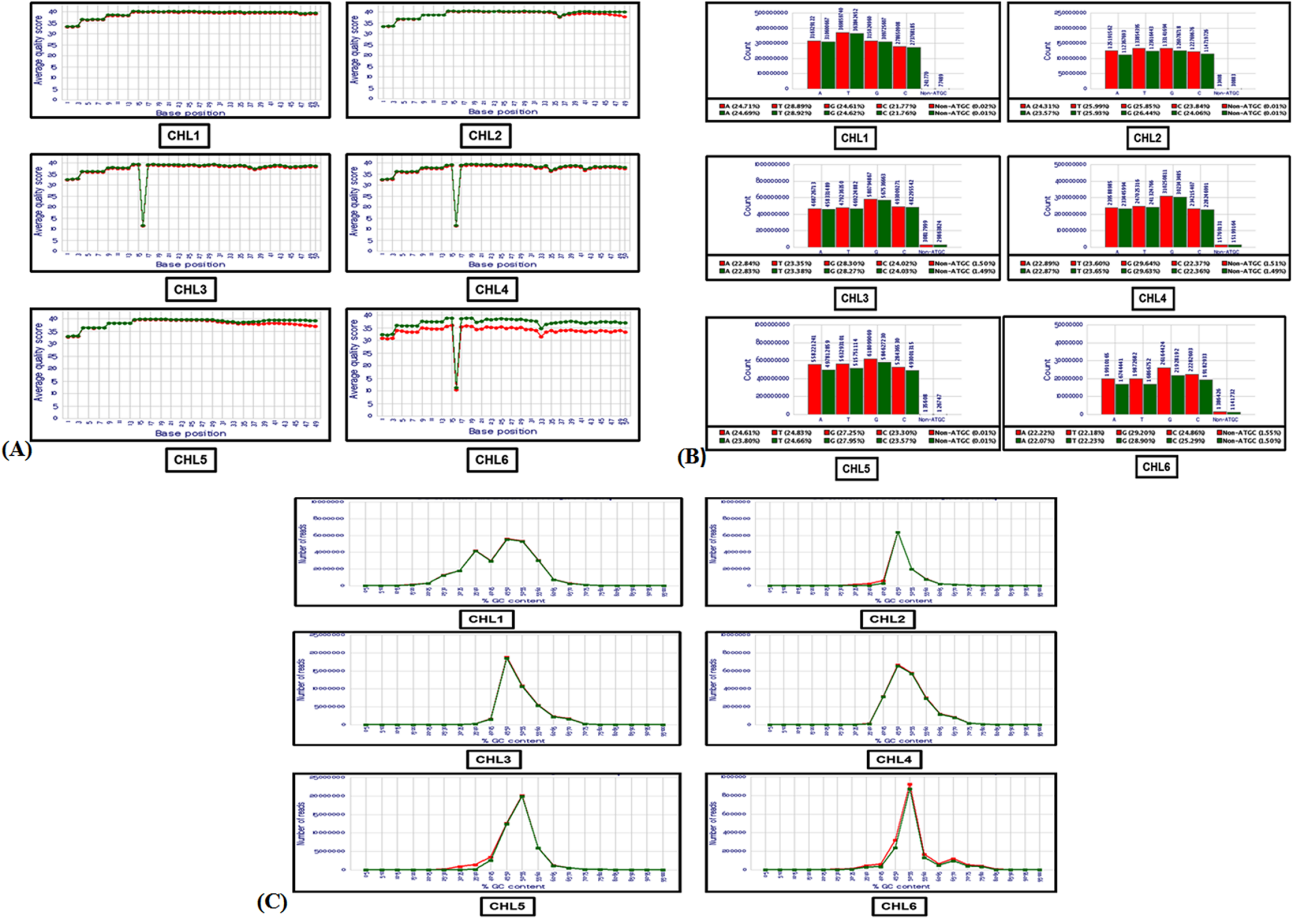
Quality assessment of small RNA reads of cardiac libraries. Quality of the NGS datasets was checked using quality assessment/quality control (QA/QC) tools. **(A).** Average quality score per position in all the raw (red) and filtered reads (green): base position is on the horizontal axis, while quality scores are on the vertical axis. **(B).** Base composition per position in all the sequencing reads (Raw/Filtered) obtained from cardiac libraries. Base positions are on the horizontal axis, while the fraction of each base is on the vertical axis **(C).** Percentage of GC content in total and filtered reads. Samples are labeled along the horizontal axis and the GC percentage is quantified on the vertical axis.

To determine the role of miRNAs in heart development, six small RNA heart libraries (CHL1-CHL6) were generated from 4^th^ to 14^th^(HH24 to HH40)day of chick embryonic stages representing fully functionalfour chambered heart development including differentiation of numerous cell types (cardiomyocytes, vascular cells, neural cells, and cardiac fibroblasts), general growth of the heart, cardiac looping,chambers formation, ventricular bending, mitral and tricuspid valve formation,differentiation of the network of Purkinje fibers, coronary veins and arteries formation. These RNA libraries were sequenced using Illumina platformHiseq 2000. In total 25,602,130 raw reads in CHL1 (4^th^ Day library); 10,510,015 raw reads in CHL2 (6^th^ Day); 41,051,704 raw reads in CHL3 (8^th^ Day);20,936,993 raw reads in CHL4 (10^th^ Day); 46,289,501 raw reads in CHL5 (12^th^ Day) and 17,923,260 raw reads in CHL6 (14^th^ Day) were obtained. The reads were filtered for low quality sequences, adapters, ribosomal RNA (rRNAs) and transfer RNAs (tRNAs) (Fig 1). The filtered datasets contained 25,161,079 clean reads in CHL1 (98.27% of raw reads), 9,730,887 in CHL2 (92.58%of raw reads), 40,150,673 in CHL3 (97.80%of raw reads), 20,413,633 in CHL4 (97.50%of raw reads), 42,679,985 in CHL5 (92.20%of raw reads) and 1,517,744 in CHL6 (84.60%of raw reads). The standard quality parameters such as base quality score, base percentages and GC% of the reads were measured. The average quality score (Q Score) was computed for each library and found to be in range of 35–40%. Each library sequences having average quality score less than 20 were not considered for further analysis. We found comparable results for raw and filtered datasets (Fig 2A). No significant deviation for A/T or G/C ratio was observed and difference between composition of A & T and G & C was less than 10% in all the stage specific heart library datasets indicating high quality of the reads. Further, we did not observe any base bias or other issues with the quality of our datasets (Fig 2B).

**Fig 3:**
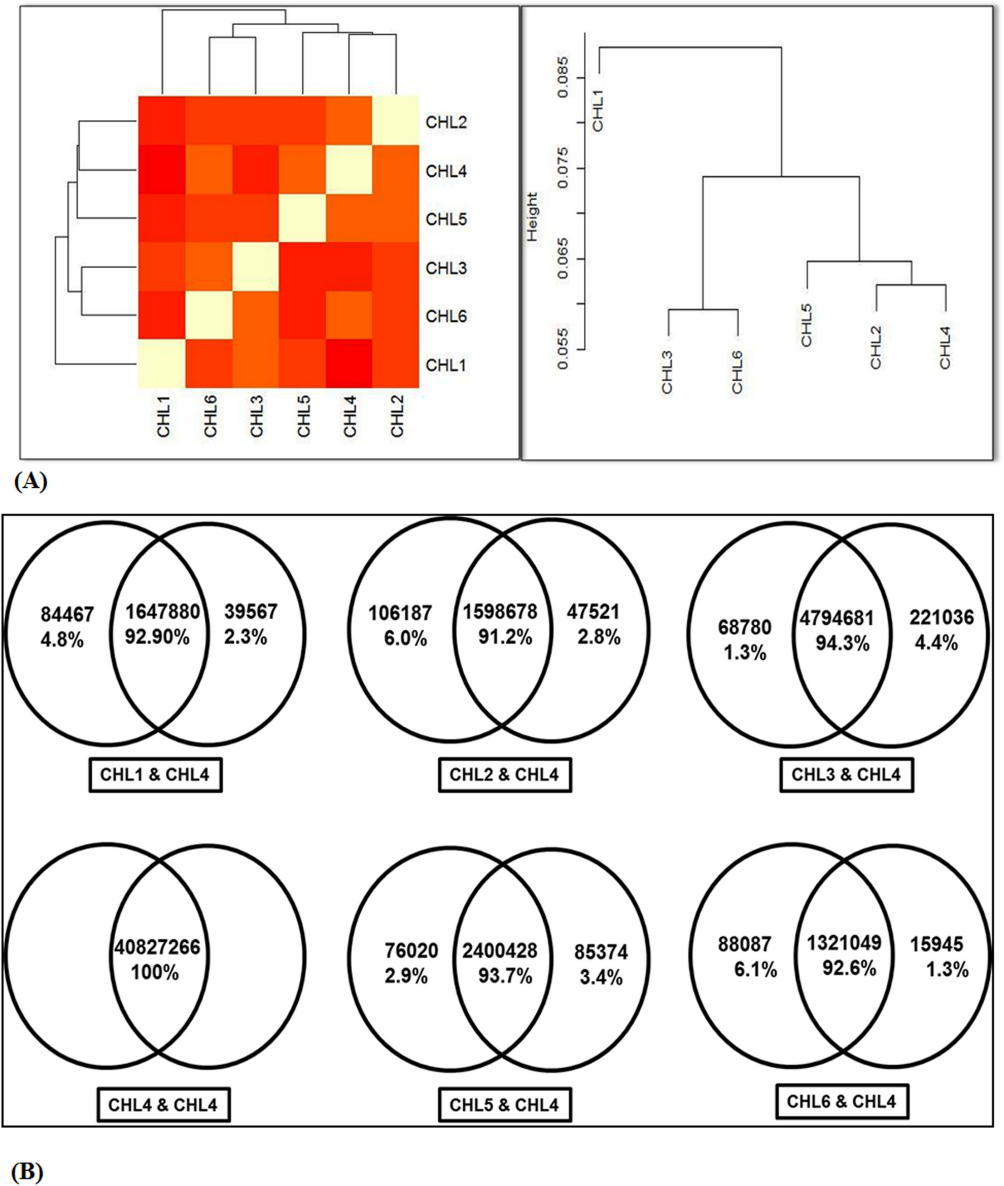
Pairwise comparison. **(A).** Hierarchical clustering of cardiac stage specific libraries. Reads obtained from all the libraries were analysed for sequence similarities. Increase in color intensity from white to red indicates the decrease in the sequence similarities. Short distances between consecutive genes on the chromosome imply that they are clustered. **(B).** Representative figure. To show the comparison of total filtered small RNA reads of CHL4 with other cardiac libraries. The overlapping portion reflects the common sequences among the libraries. The peculiar percentage in libraries showed their respective unique sequence reads.

**Fig 4:**
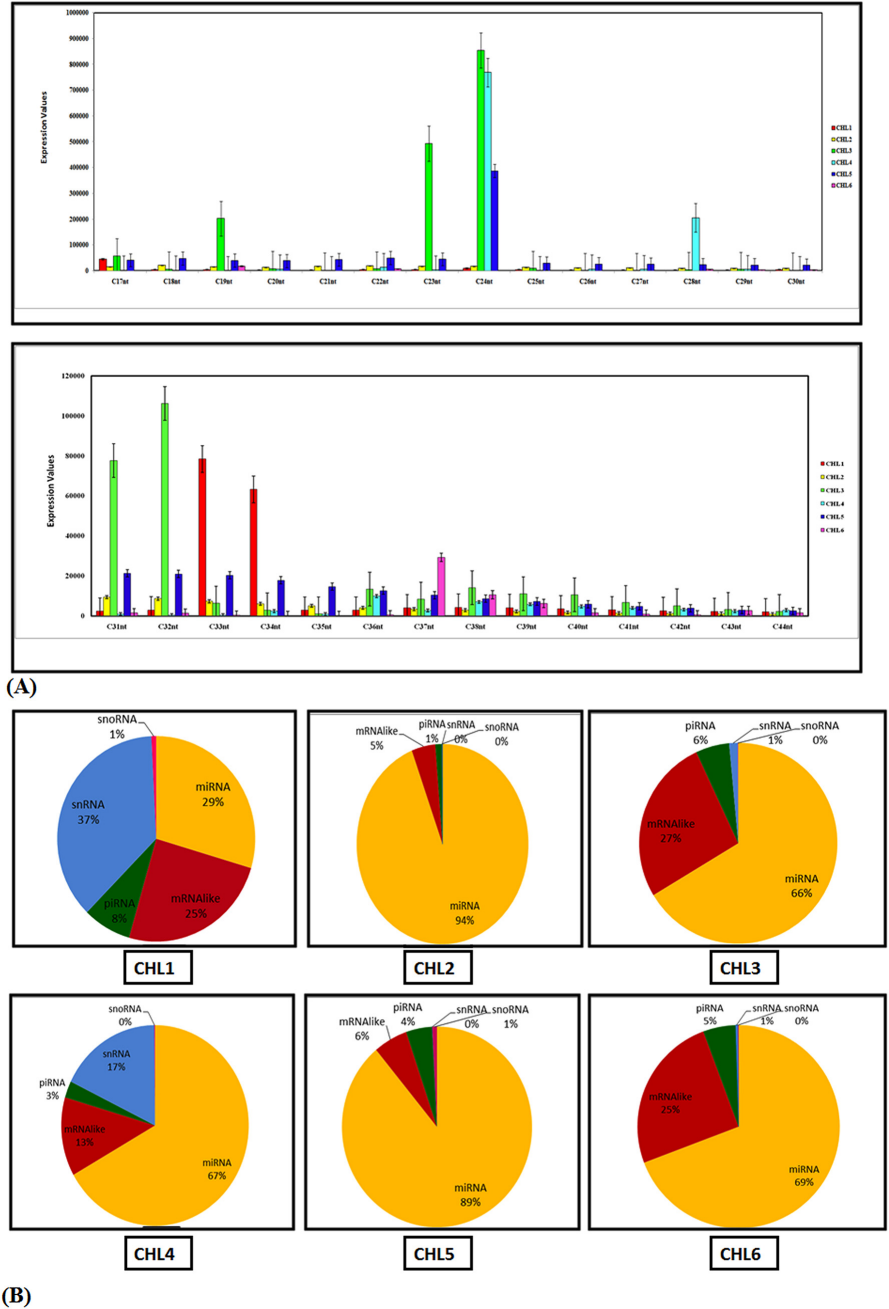
Characterization of small sequence reads of all six libraries. **(A).** Total small read lengths distribution. Read length (in base pairs) is on the horizontal, while the number of reads is on the vertical axis. **(B).** Total small RNA type distribution. Annotation of small RNA species to analyse the percentage microRNA populations and other classes of RNA species in all six cardiac datasets.

Next, GC% in all the library reads was examined for quality assurance. We observed that all the raw and filtered library sequences had similar GC content.Overall GC content did not differ significantly between six datasets. Only CHL1 dataset had a bimodal distribution of GC content with a prominent peak at 50% and a second peak at around 40%. All other libraries datasets reflected unimodal GC pattern showing a prominent peak at 50% GC. However CHL4 had a broader GC distribution as compare to others (Fig 2C). GC content of all libraries ranged between 45–55% that matched with GC% of chicken genome, confirming the authenticity of library datasets. In addition, we performed pair wise sequence alignments of total small RNA reads (common and stage specific) of all six libraries (Fig 3A). Weobserved that stage/library specific population occupied 5% to 15% of whole dataset suggesting presence of stage specific small RNAs during the progression of heart development (Fig 3B). The overall size distribution of all sequenced reads across different libraries were similar, with the 24nt class being the most abundant, followed by the 23nt showing bimodal pattern (Fig 4A). The length of majority of the members of library were in the range of 20 to 34nt. CHL3 and CHL5 had more small RNAs of diverse read length, while other libraries had limited length of small RNAs.

Subsequently, the small RNA sequences were mapped and searched against SnRNAs, SnoRNAs, piRNAs, miRNA like and miRNAs. Sequences shorter than 18nt and longer than 28nt were not considered for further analysis. We found that all the libraries were enriched in microRNAs sequences. miRNAs sequences accounted for 29% of whole small RNA population in CHL1 library, whereas occupy 94% of CHL2 population, 66% of CHL3, 67% of CHL4, 89% of CHL5 and 69% of the small RNAs of CHL6. The differences in the distribution further suggested the differential expression of microRNAs at various stages of heart development. CHL2 (6^th^day) and CHL5 (12^th^day) had highest percentage of microRNA sequences (94% & 89% respectively) while CHL1 (29.6%) had least abundant microRNA population indicating role of miRNAs w.r.t. heart development (Fig 4B). We further subjected microRNA sequences for detailed characterization.

### Identification of MicroRNA during cardiogenesis

**Fig 5:**
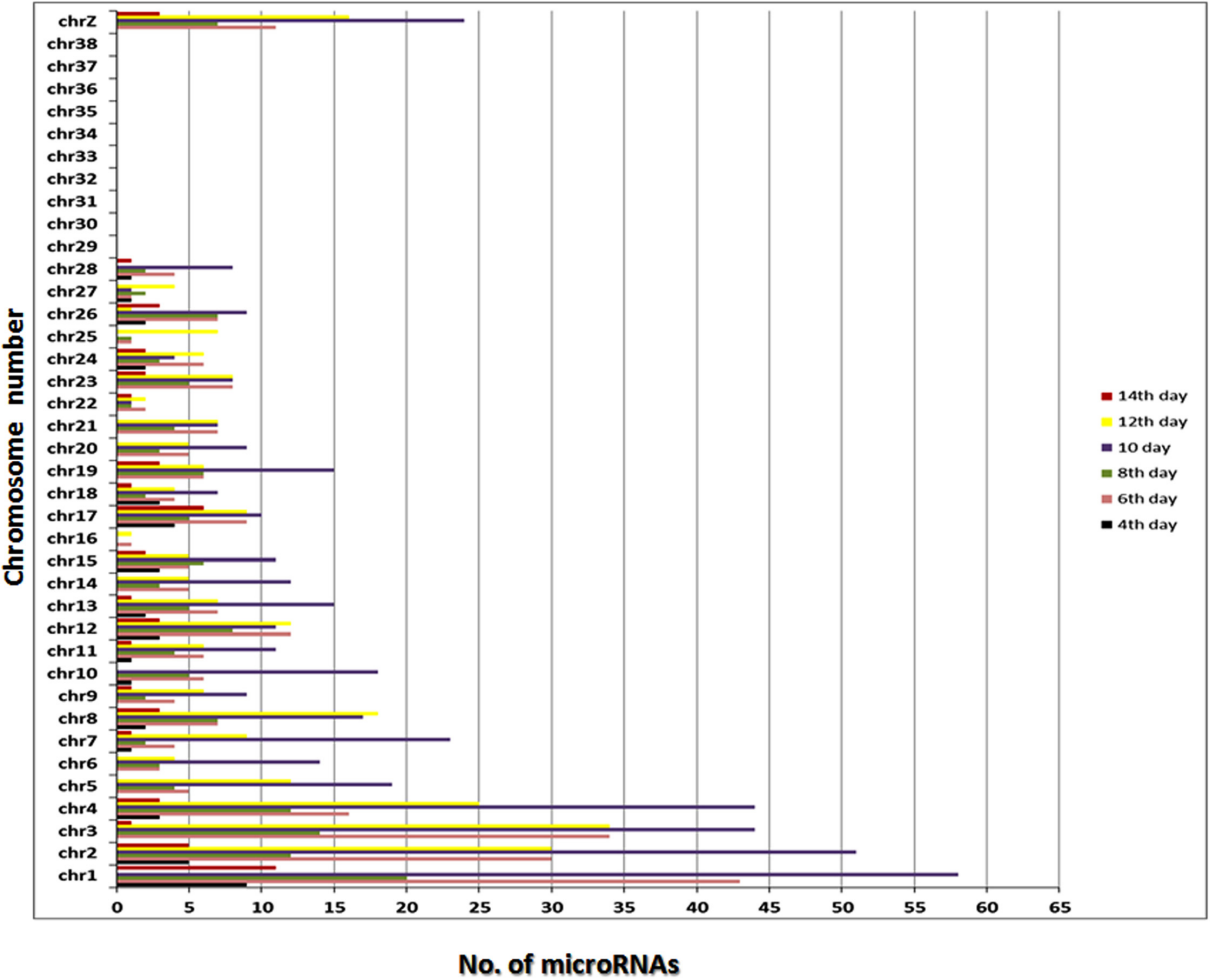
Chromosomal distribution of cardiac specific microRNAs. miRNA precursor sequences from all libraries were searched extensively in miRBase to find their location in different chicken chromosomes. The histogram depicts the variations in the distribution of miRNAs in different chromosomes of *Gallus gallus* among the libraries.

**Fig 6:**
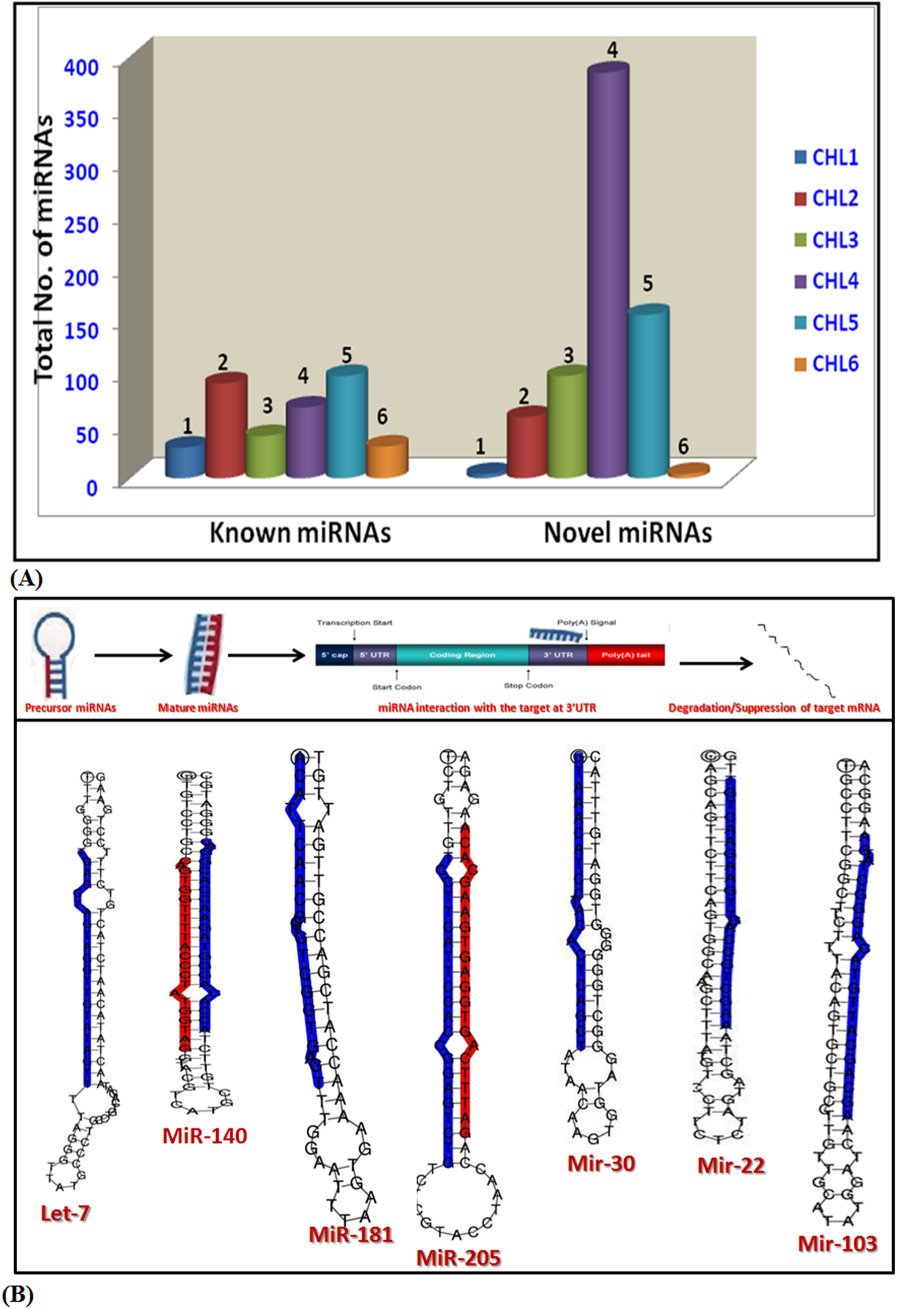
Characterization of microRNA sequences. **(A).** Identification of microRNAs. Extracted miRNAs and their precursor sequences from all the libraries were compared with the mature chicken miRNAs of miRBase 19.0 allowing zero mismatches. Consequently known and novel microRNAs were obtained from all the stage specific cardiac libraries. Histogram shows the variation in the total number of microRNAs among the libraries. **(B).** Precursor structure of cardiac specific microRNAs. Precursors of all the known and novel miRNAs were predicted by analysing the typical stem–loop structure in the flanking regions of miRNA genes. Folded structures of precursors of seven conserved microRNAs identified in all six libraries are shown for representation.

To comprehend the distribution pattern of all the miRNA reads on chromosomes (chr), the location of each tag in all the stage specific cardiac libraries on chicken genome was analysed and a chromosome wide distribution map was created for all the miRNA reads. We observed differential distribution patterns of miRNAs in different libraries that correlated with the size of the chromosomes with some exceptions. chr 1 was found to harbors maximum number of miRNAs followed by chr 2, chr 3 and chr 4 respectively among the libraries (Fig 5). miRNAs were searched in mirBase database to identify conserved miRNAs across the six libraries.We found that 353 (33.4%) miRNAs were already known and 703 (66.6%) were novel microRNAs in our datasets(Fig 6A). Maximum microRNA population was found to be expressed on 10^th^ day of developing chicken heart (CHL4), whereas, the early (CHL1) and late developing stage (CHL6) had least number of expressed microRNAs suggesting differential involvement and spatio-temporal expression of miRNAs and their possible targets.

**Fig 7:**
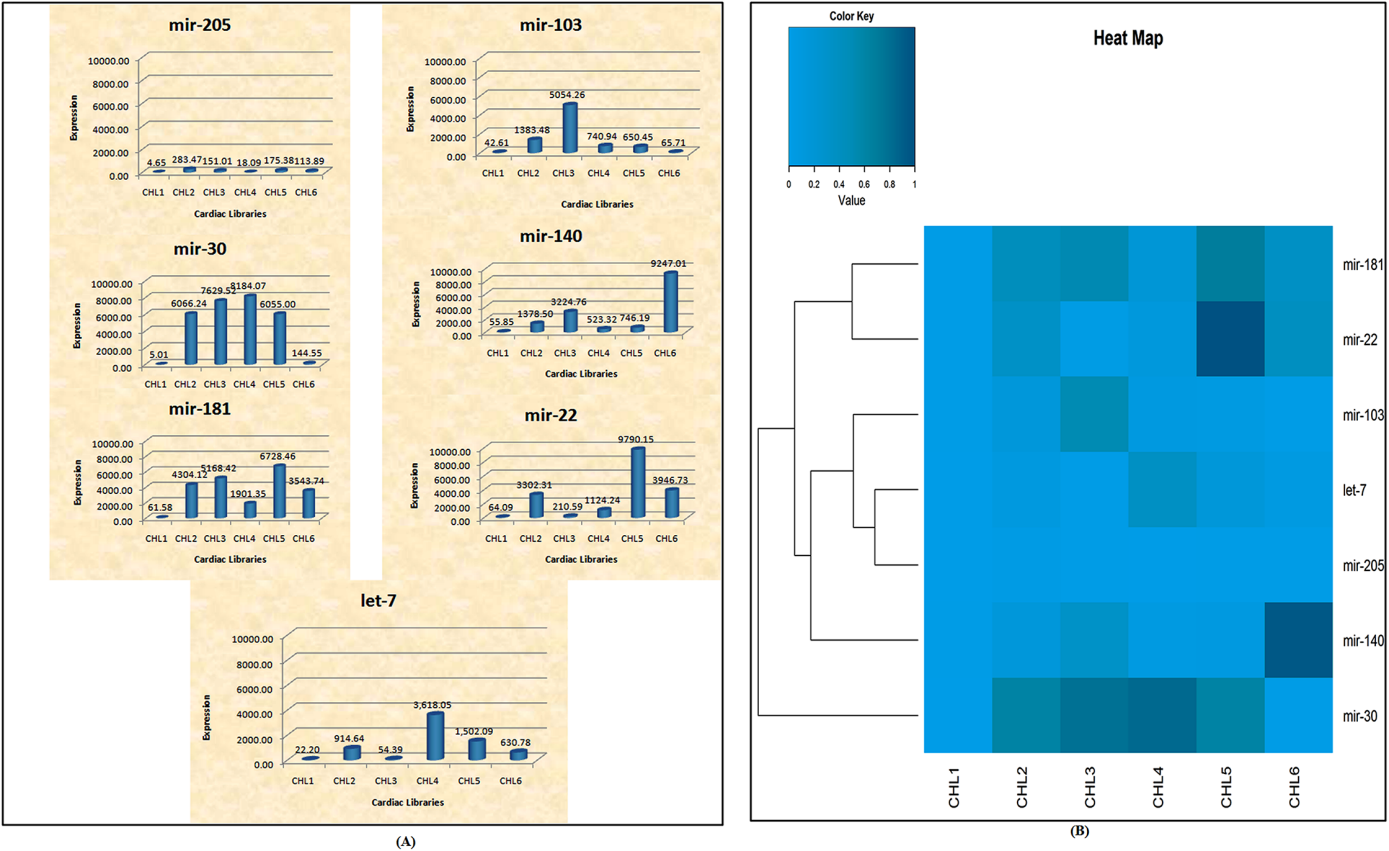
Expression analysis of conserved microRNAs in all six libraries. **(A).** Real time PCR studies: the relative abundance/expression of seven conserved microRNAs (3D view). **(B).** Heat maps of microRNAs expression profiles. Fold change of seven microRNAs that were conserved among the libraries based on deep sequencing data. Box represents the color coding of the heat map with the numbers indicating relative signal intensity.

The detection of novel miRNAs in our study raised the number of unique miRNAs in literature from 791 to 1494. Out of total miRNAs identified, a total of 32.7% were known miRNAs (termed DCmirs) which displayed differential expression and 0.6% were conserved known microRNAs (termed CCmirs). Seven well known miRNAs namely let–7, miR–140, miR–181, miR–30, miR–205, miR–103 and miR–22 have been found to be conserved in all the stages of chick heart development as well as conserved among model organisms including fly, human, chicken, rat and mouse.These seven conserved miRNAsvery well correlates with the developmental stages and listed in GEISHA, a chicken embryo gene expression database and are predominantly enriched in cardiac and skeletal muscle tissues during the development.Our deep sequencing-based genome and transcriptome analysis confirmed the presence of unique miRNA sequences in all the libraries (data not shown). About 66.5% of total dataset identified in our study was unique and not reported earlier, therefore categorized as novel stage specific cardiomiRs (NSSCs). The unique sequences of NSSCs from each library were aligned with the chicken genome and transcriptome. Following parameters were used to qualify a miRNA as unique or novel in nature: *(i)* sequences length should be of 18 to 30nt, *(ii)* Upto 18 kcal/mol was allowed as maximum free energy to identify miRNA precursor, *(iii)* maximum space between miRNAs and miRNAs* was 35nt, *(iv)* maximum bulge of miRNA and miRNA* was 4 and *(v)* secondary folded structure of the potential miRNAs precursors predicted by microRNAfold using MFE RNA structure prediction algorithm.All the unique miRNA sequences fulfilled these conditions and were considered as bonafide novel miRNAs. Thereafter, folded hairpin loop structures were checked for all three classes namely CCmirs, DCmirs and NSSCmirs (Fig 6B). We performed the expression analysis of CCmirs which revealed stage specific modulation of cardiac microRNAs (Fig 7A). CHL1 had least expression of all seven CCmirs. Specifically, miR–30 expression studies revealed an increased expression during heart development (CHL1-CHL4) but it was reduced in fully grown heart (CHL5) and had least expression in CHL6, where heart attained its adult phenotype. This data suggested that miR–30 can serve a potential regulator of fetal cardiac gene program. Heat maps were also generated for all the known and novel microRNAs in all six stages of heart development to observe their expression (Fig 7B, S2 and S3 Figs).

### Prediction of cardiac microRNA specific targets

To assess the biological meaning of the chicken cardiac miRNAs and their target genes, we examined representation of GO terms in the whole set of target genes and within each of the six libraries. We predicted the potential targets and estimated interspecies conservation of known and novel miRNA sequences using Pic Tar, Target Scan and miRanda algorithm.

**Fig 8:**
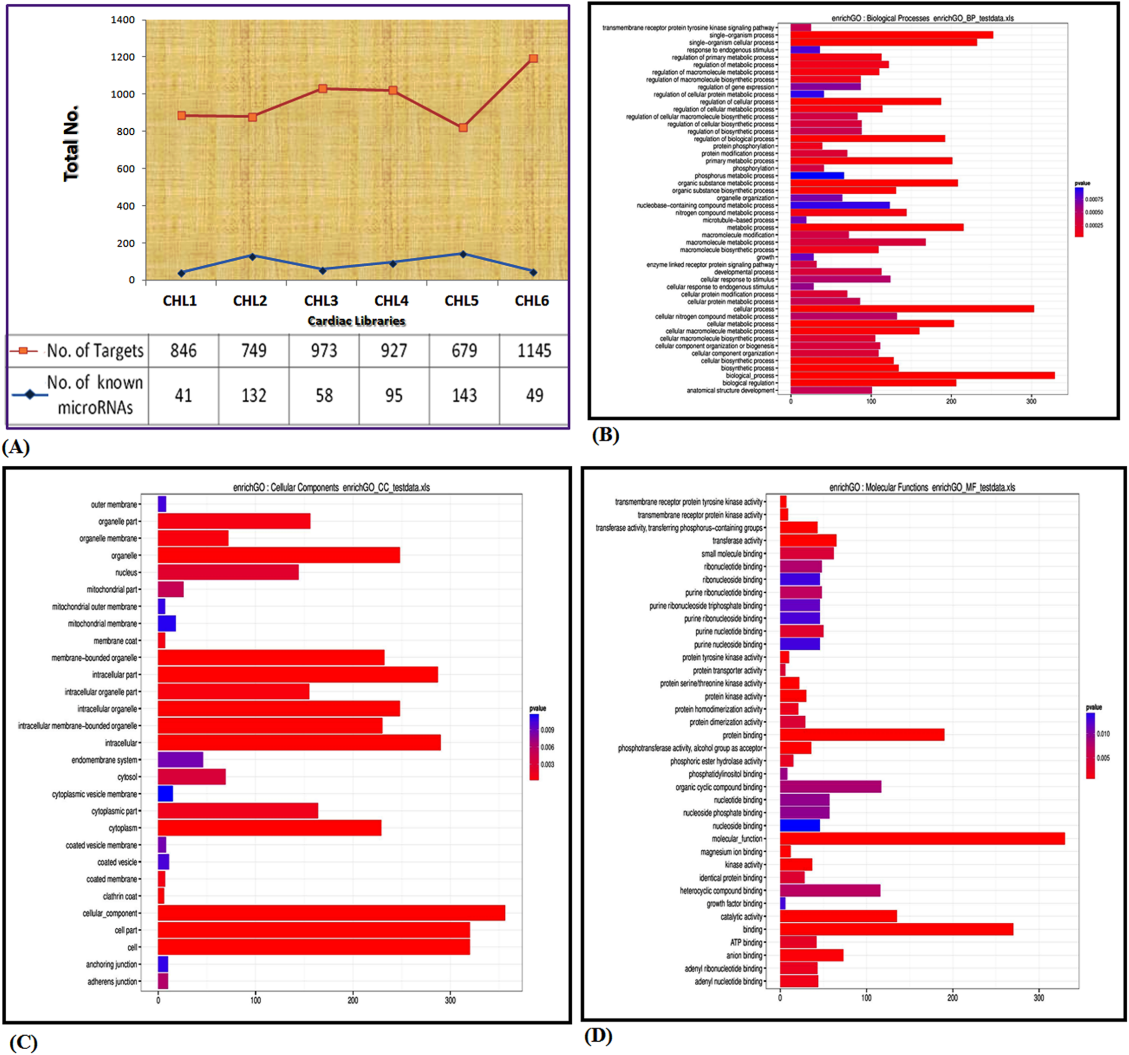
Characterization of targets. **(A).** Prediction of microRNA targets. Figure represents the total number of microRNAs (blue trend line) and their targets (red trend line) in all six stage specific cardiac libraries. **(B).** Gene ontology studies for *biological processes* annotation of predicted target genes of total microRNAs **(C).** Gene ontology studies for *cellular components* **(D).** Gene ontology studies for *molecular functions*. (CHL4 was taken as a representative candidate heart library).

In our work, we found several targets for different miRNA families spread across various libraries. In Fig 8A, the frequency of known miRNAs showed a 3.5 fold difference between 4^th^ day and 12^th^ day of heart development. Whereas, there was 1.5 fold difference in number of targets corresponding to known miRNAs on 4^th^ day and 14^th^ day suggesting weak/strong correlation.

**Fig 9:**
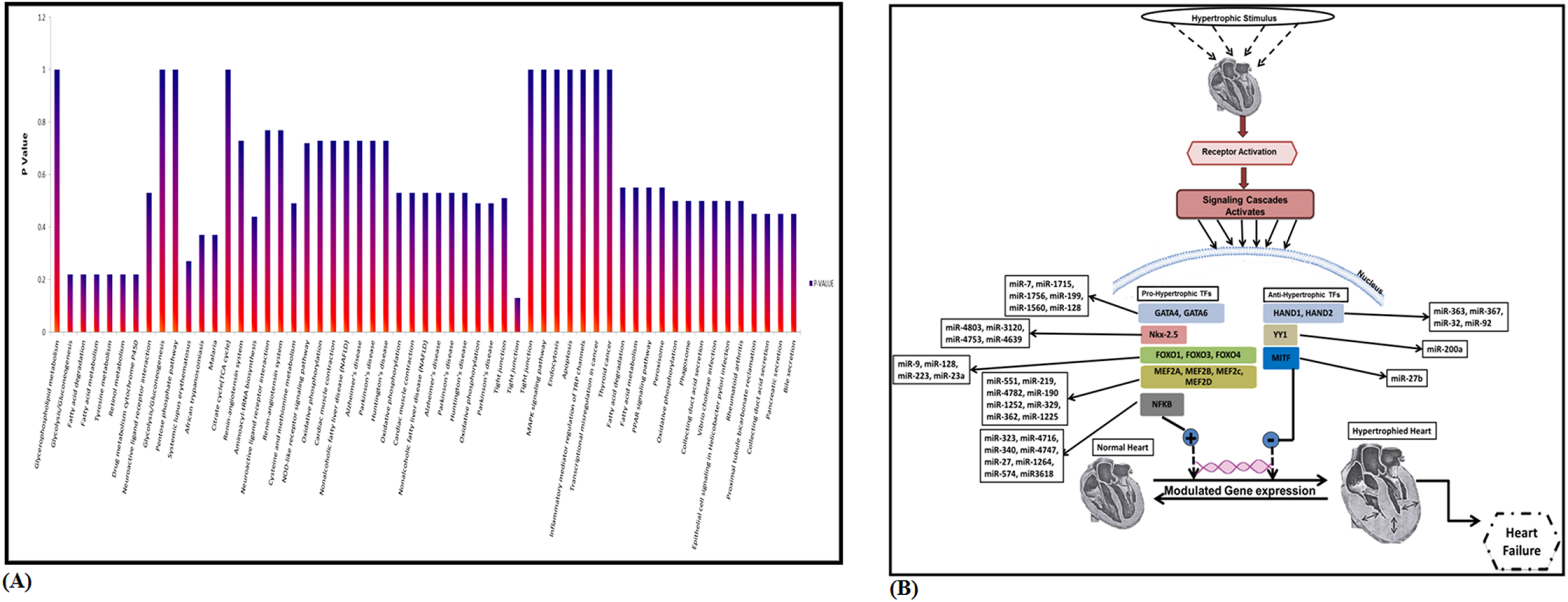
Pathway involvement. **(A).** KEGG pathway enrichment analysis of cardiac specific target genes identified in Cardiac library (CHL4 as representative candidate library). Only significantly KEGG functional categories (p < 0.05) were depicted according to their p-value. **(B).** miRNAs-transcription factor interactions. Hypertrophic stimulus leads to activation of multiple signalling pathways which converge on to a common program targeting the activity of cardiac transcription factors and reactivation/modulation of fetal/cardiac gene program in the nucleus which manifest finally as the hypertrophic phenotype. Figure represents that both pro and anti-hypertrophic TFs were targeted by known miRNAs identified in all six libraries.

The target mRNAs from all six libraries were subjected to GO analysis (S2 Table). For example, we predicted 619 gene targets for differentially expressed miRNAs obtained from CHL1 library. Functions of 60% of predicted gene targets of differentially expressed miRNAs were mapped to cardiac system. There was 130 fold enrichment of growth & development related GO terms such as system development, morphogenesis, organ development, cell differentiation, cell growth, regulation of growth, embryonic development, regulation of development and pattern specification. Similarly, we found over 400 target gene families for miRNAs belonging to CHL4 and 80% of these genes were present in first sub-category, *biological process*. Out of these, 111 genes (33.73%) were found in cellular component organization & biogenesis, 87 genes in regulation of gene expression (26.44%), 70 genes in protein modification process (21.27%), 29 genes in embryo development (8.8%), 10 fibroblast growth factor receptor signaling pathways (3.03%) and many other biological process (Fig 8B, S3 Table). The second sub-category, *cellular process* includes 86.82% dataset of target genes in which 248 (69.66%) genes were predicted to be included in intracellular organelle, 232 (65.1%) genes in membrane bound organelle and 26 (7.30%) genes in mitochondrial membrane (Fig 8C, S4 Table). The third sub-category, *molecular function* includes 80.48% of target genes, out of which 190 genes were involved in protein binding (57.05%), 135 genes in catalytic activity (40.90%), 84 gene in protein kinase activity (25.40%) and 57 genes in nucleotide binding (17.27%) (Fig 8D, S5 Table). Similarly, we performed GO analysis for remaining libraries also (data not shown).To understand the heart specific expression of predicted targets, we searched the targets in reported literature as well as in Gencard, a database for all known and predicted human genes where microarray and illumina platform based embryonic tissue specific expression data is listedand found significant enrichment of heart and development related target genes strengthening the concept of involvement of miRNAs-mRNAs/targets for coordinating heart development (S4 Fig, S6 Table).We attempted to predict metabolic pathway enrichment using *KAAS* tool (http://www.genome.jp/tools/kaas) and observed overrepresentation of 11 pathways (Fig 9A, S7 Table). The molecules and pathways were involved in muscle development, fibroblast contraction, cardiac muscle contraction, adrenergic signaling in cardiomyocytes, and vascular smooth muscle contraction. Interestingly, most of the pathways have been shown to be involved in the growth and developmental processes, including the skeletal muscle development. For example, in the top five enriched pathways, the MAPK pathway was present which can regulate a wide variety of cellular functions, including cell proliferation, differentiation, and stress response. We also observed the presence of apoptotic pathway, which is known to play a central role in controlling embryonic development in different organisms ranging from chicken to human. Further, recent studies show that apoptosis signaling induces myoblast differentiation and regulate myocardial stress responses. Interestingly, a substantial fraction of transcriptional regulators were targeted by microRNAs obtained in our study. Many pro and anti-hypertrophic cardiac transcription factors (TFs) such as GATA4, MEF2, NKx2.5 and SRF etc. were the targets of cardiac specific microRNAs (Fig 9B), suggesting the combinatorial interaction of miRNAs-TFs in regulation of cardiac fetal gene programs.

## Discussion

Our study demonstrates utility of combining next generation sequencing technology and experimental techniques with bioinformatics approaches to address several questions relevant to cardiac development and diseases. To our knowledge, this is the first study conducted to catalogue the complete set of microRNAs expressed during the heart development and differentiation.

Chicken is considered to be a model organism to study cardiac development and diseases due to extensive conservation of pathways and genes with humans [46]. The study is important owing to the fact that cardiogenesis requires delicate and controlled regulation of gene expression and defective regulation of fetal gene program may lead not only to congenital heart diseases, the most common birth defects but also many adult cardiac abnormalities which involve re-expression of fetal gene program and have developmental basis [47]. Cardiac hypertrophy and heart failure are the most common pathological responses to several cardiovascular diseases where fetal gene programming is believed to be the critical marker. Normally, fetal genes express during heart development, but are silenced after birth [48]. However, their expression is re-induced in the ventricular myocardium in response to cardiac stress. Thus, understanding the molecular mechanisms that govern the expression of fetal cardiac genes could lead to the discovery of novel regulators involved in both cardiac development and diseases. microRNAs in that regard, has emerged as critical regulators for the differentiation and growth of cardiac cells, and it is therefore reasonable to hypothesize that fetal miRNAs may play important roles in cardiac hypertrophy and heart failure [49]. Several miRNAs such as miR–1, miR–126, miR–133, miR–138, miR–208, miR–290 and miR–302 have emerged as critical regulators for the cardiac patterning, conduction system, cardiogenesis, angiogenesis, cardiac hypertrophy and heart failure [50].

In the present study, we constructed heart specific libraries instead of using whole embryo to identify microRNAs specific to cardiac development. This approach led to the identification of several known (353) as well as novel (703) microRNAs. Together with the previously reported set of total 791 known miRNAs from chicken [51], this brings the total number of miRNAs in the chicken to 1494. Our study showed that more than 1000 microRNAs were found to be significantly involved in cardiac growth, development and differentiation. Majority of them are not annotated and many of these are unique and conserved to primate species. We observed both conserved as well as differentially expressed miRNAs in the cardiac tissues of various developmental stages. Our results indicate that more than 500 known miRNAs showed differential and stage-specific expression. Relatively, few miRNAs were detected during early cardiogenesis. Our data is in agreement with previously reported miRNAs in context of heart development, further suggesting the role of microRNAs towards the formation of complex heart having numerous cell types and differential expression of specific set of genes. It may be hypothesized that during the heart development and diseases, the differentially expressed miRNAs may regulate both the spatial-temporal expression of genes and levels of the target mRNA expression, asdemonstrated previously in few studies [52]. Alsoone of the potential biological applications of this unique expression pattern of miRNAs would be contribution to understanding of heart development. On 4^th^ day of chick heart development- atrial septation leads to obliteration of the interatrial communicationand cardiac looping processing occurs. Further, on 5^th^ day formation of coronary vasculature and epicardium begins. Therefore, we can suggest that presence of high expression of miR–26, miR–10 as well as miR–92 seems to be important in anatomical orchestration of chicken heart. The 6^th^ day is marked by the formation of ventricle septation and valve development which could be correlated with increased expression of miR–30, miR–92, mir–10 and mir–26. The 8^th^ day is characterised with development activities namely completion of ventricular septation, outflow tract septation, coronary vasculature developments, endocardial cushions, myocardial proliferations cardiac innervations and presence of apoptotic cells around branch point of great vessels. At miRNA expression level, we observe expression of miR–126, mir–140, mir–146, mir–30, mir–454, mir–103, and mir–301 in CHL3 library. Similarly, several miRNA shows increased and distinct expression on day 10^th^, 12^th^ and day 14^th^ of chick heart development. The diverse distribution as well as expression pattern of cardiac specific microRNAs strongly support the concept microRNAs function at all levels to regulate developmental processes.

To understand more about our observations in context of fetal gene associated heart diseases, we compared our data with previously published studies and observed significant overlap with the list of miRNAs reported earlier in the context ofheart diseases. For instance, there were 47 miRNA reported in context of delayed cardiomyopathy and we observed 22% to 39% overlap with miRNA expressed at various stages of heart development. Similarly, we found overlap of miRNA population reported in conditions like heart failure and cardiac fibrosis (S8 Table). This suggests that miRNAs expressed during early stage heart development have potential role during diseased state in humans. This also points to possible reasons of re-expression of fetal genes during terminal stages of heart diseases in humans. Among the known cardiac microRNAs obtained in our study, let–7 family, the second microRNA found in *C*. *elegans*, have recently been found to be highly expressed in the cardiovascular system [53]. Studies have revealed the aberrant expression of let–7 members in cardiovascular diseases, such as cardiac hypertrophy, cardiac fibrosis, dilated cardiomyopathy, myocardial infarction, arrhythmia, angiogenesis, atherosclerosis and hypertension [54]. Similarly, miR–122 over expression is associated with induced cardiac myocytes apoptosis, an important feature evident during heart failure [55]. miR-10a regulates proliferation of human cardiomyocyte progenitor cells by targeting cardiac transcription factor GATA6 [56]. miR–100 and miR-133b regulate the reprogramming of key remodelling /fetal genes which are involved in the establishment and progression of common human cardiomyopathy [57]. miR–138 regulates the cardiac patterning which are highly conserved from zebrafish to Humans [58]. miR–126 is a key positive regulator of angiogenic signaling and vascular integrity [59]. miR–22, miR–23, miR–30, miR–125, miR–103, miR–181, miR–199, miR–20, miR–126 have been reported to be over expressed in human failing heart [60]. Considering the same miRNA expression and possibly the involvement of their respective gene targets, it appears that similar mechanisms are at play in heart failure and heart development by the re-expression of fetal gene program [61]. Computational prediction revealed more than 1000 putative target genes for cardiac specific microRNAs. The targets appeared to be involved in a broad range of biological processes with most of the genes related to cell cycle, apoptosis, signaling pathways, extracellular remodelling and gene regulation. Majority of the targets have significant role in heart development and diseases [62–65].

The future goal of the study is to design the microRNA based therapeutics against cardiomyopathies where hypertrophy in terminally differentiated adult cardiac myocytes is witnessed. Cardiac hypertrophy is an adaptive response to pressure or volume stress which accompanies many forms of heart disease, including hypertension and heart failure [66]. During hypertrophy, myocytes increase in size along with enhanced protein synthesis, sarcomereric re-organization and re-expression of fetal genes such as atrial natriuretic peptide (ANP), B-type natriuretic peptide (BNP) and beta-myosin heavy chain (β-MHC) [67]. Existing anti-hypertrophic drugs including beta blockers, Angiotensin converting enzyme (ACE) inhibitors etc. target the outside-in signalling pathways only. Due to complexity of pathophysiology and cross-talk between the complex signaling pathways implicated in cardiac development and diseases [68], existing therapies have limited success in controlling heart diseases. In our opinion, for a therapy to be successful, nuclear regulators should be viewed as potential therapeutic targets for designing the anti-hypertrophic drug as all the signals eventually converge on a common cardiac gene activation program. In this regard, miRNA and cardiac transcription factors (TFs) should be taken in to consideration for future study to design successful therapies against hypertrophy as they are critical nuclear regulators for modulating cardiac gene program [69–70]. It would be interesting to explore miRNA-TF axis as a new line of target for drugs against cardiac hypertrophy. Understanding the combinatorial regulatory networks among cardiac TFs and miRNAs, especially tissue-specific miRNAs would represent a significant link in deciphering molecular basis of cardiac-specific gene expression during development and diseases [71–72]. In our study, many of the cardiac TFs such as GATA4, NKX2.5, HAND, MEF2, SRF etc. were the potential targets of microRNAs identified in deep sequencing. Most of TFs and miRNAs interactions have not been studied till date. Hence, our study conducted on tiny sequences has a big potential not only for understanding the developmental switch but also in generating novel therapies against fetal gene reprogramming associated with cardiac abnormalities using microRNA based approaches.

## Conclusion

Taking these findings together, the current study has uncovered 353known and 703 novel miRNAs associated with chick heart development. The GO term and KEGG pathway annotations for the predicted miRNA targets show that these microRNAs are enriched in functional categories and pathways, which play important roles in heart development. Future studies can be designed to analyse the expression pattern and to decipher miRNA-transcription factors relationship to identify potential candidates for microRNA based therapeutics against heart failure, a leading cause of mortality worldwide.

## Supporting Information

S1 Fig Schematic representation of strategy adopted for stage specific miRNA profiling and characterization. (TIF)

S2 Fig Heat maps of selected differentially expressed microRNAs expression profiles. (TIF)

S3 Fig Heat maps of few novel microRNAs expression profiles. (TIF)

S4 Fig Comparative analysis of target expression: The heat map was plotted on the basis of compared microarray and illumina gene expression data obtained from GeneCards database using ‘R’ programme. The paired T-test was done to check the significance of expression data using origin 6.1. We found that the obtained data was 95% accurate at 0.05 significant levels.
(TIF)

S1 Table List of microRNAs and their primer sequences. (DOCX)

S2 Table Summary of miRNA targets and their scores with minimum free energy, identified in six libraries CHL1 –CHL6. (XLS)

S3 Table GO analysis for *biological processes-* CHL4 targets. (XLS)

S4 Table GO analysis to understand *cellular process*es of CHL4 targets. (XLS)

S5 Table GO analysis to map the*molecular functions*of CHL4 targets. (XLS)

S6 Table Summary of KEGG analysis of CHL4 targets. (XLSX)

S7 Table List of known miRNAs having role in cardiac development and diseases (extracted from existing literature) and their comparative analysis with miRNAs identified in each library of our study. (XLS)

S8 Table List of targets identified in our study and their role in heart discussed in published literature. (XLS)
