# Supplementary figures and images for: Comparative Characterization of Cardiac Development Specific microRNAs: Fetal Regulators for Future
Source: PLoS One. 2015 Oct 14;10(10):e0139359. doi: 10.1371/journal.pone.0139359 (PMC4605649; doi:10.1371/journal.pone.0139359)

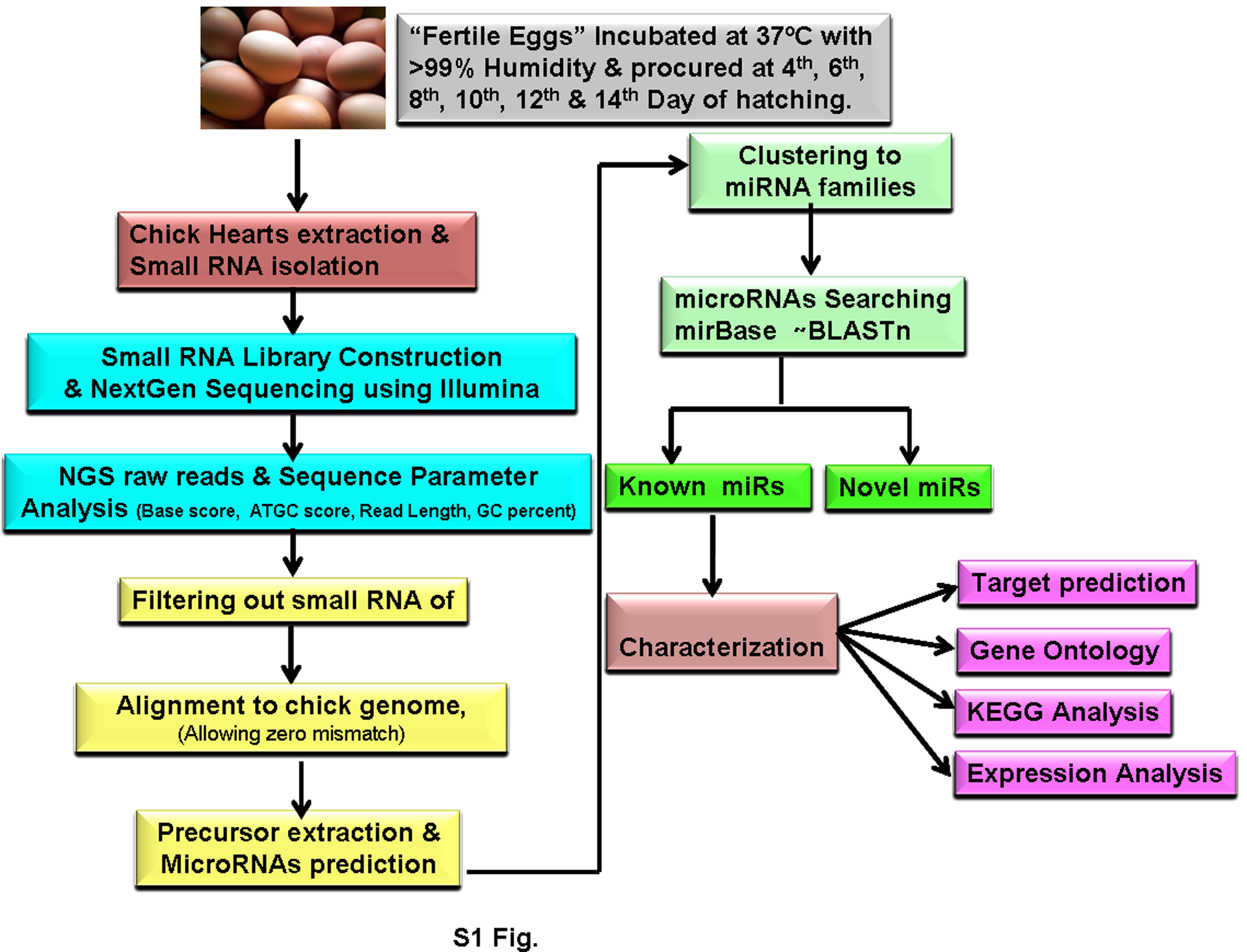

Supplement: S1 Fig — (TIF) [file pone.0139359.s001.TIF]

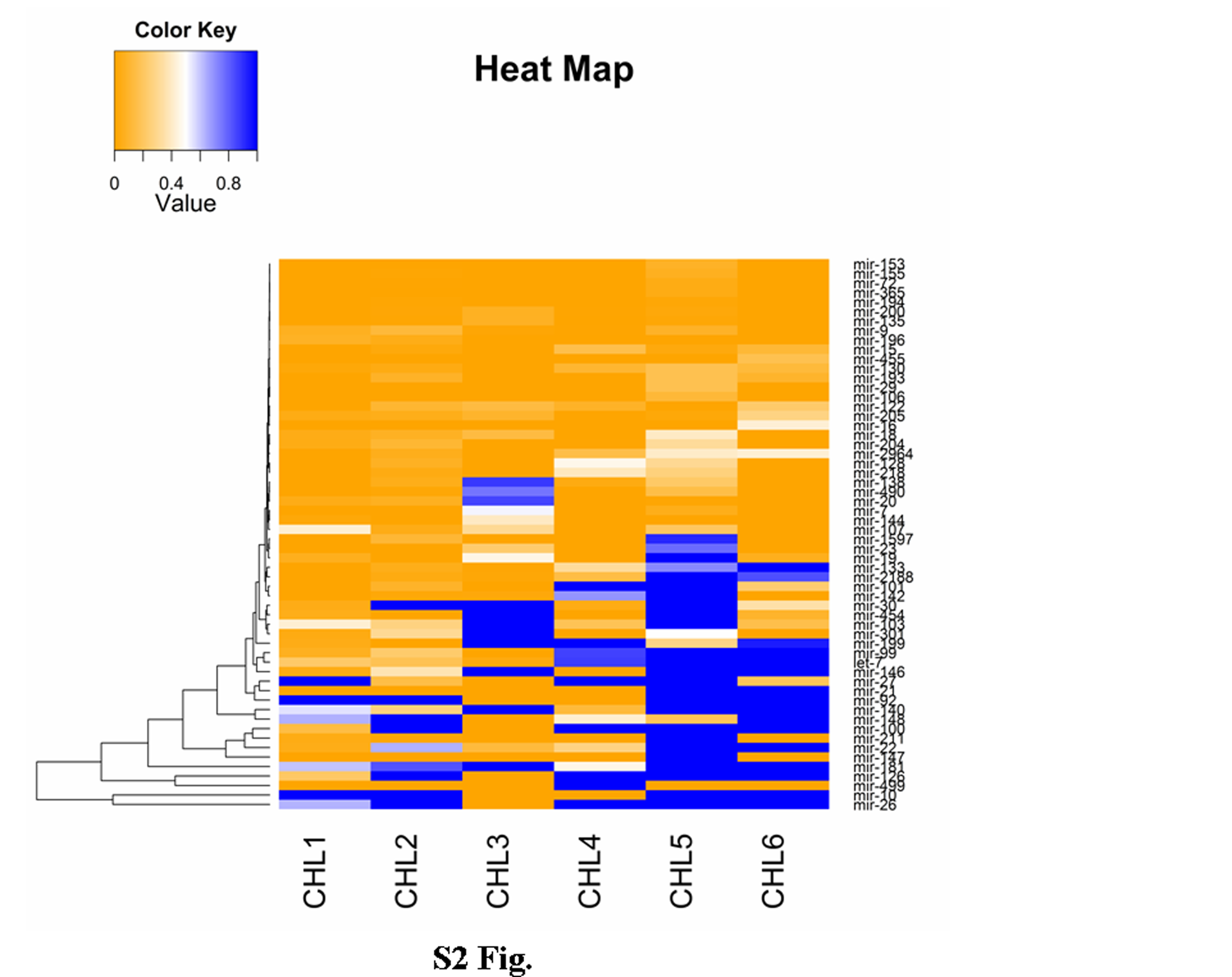

Supplement: S2 Fig — (TIF) [file pone.0139359.s002.tif]

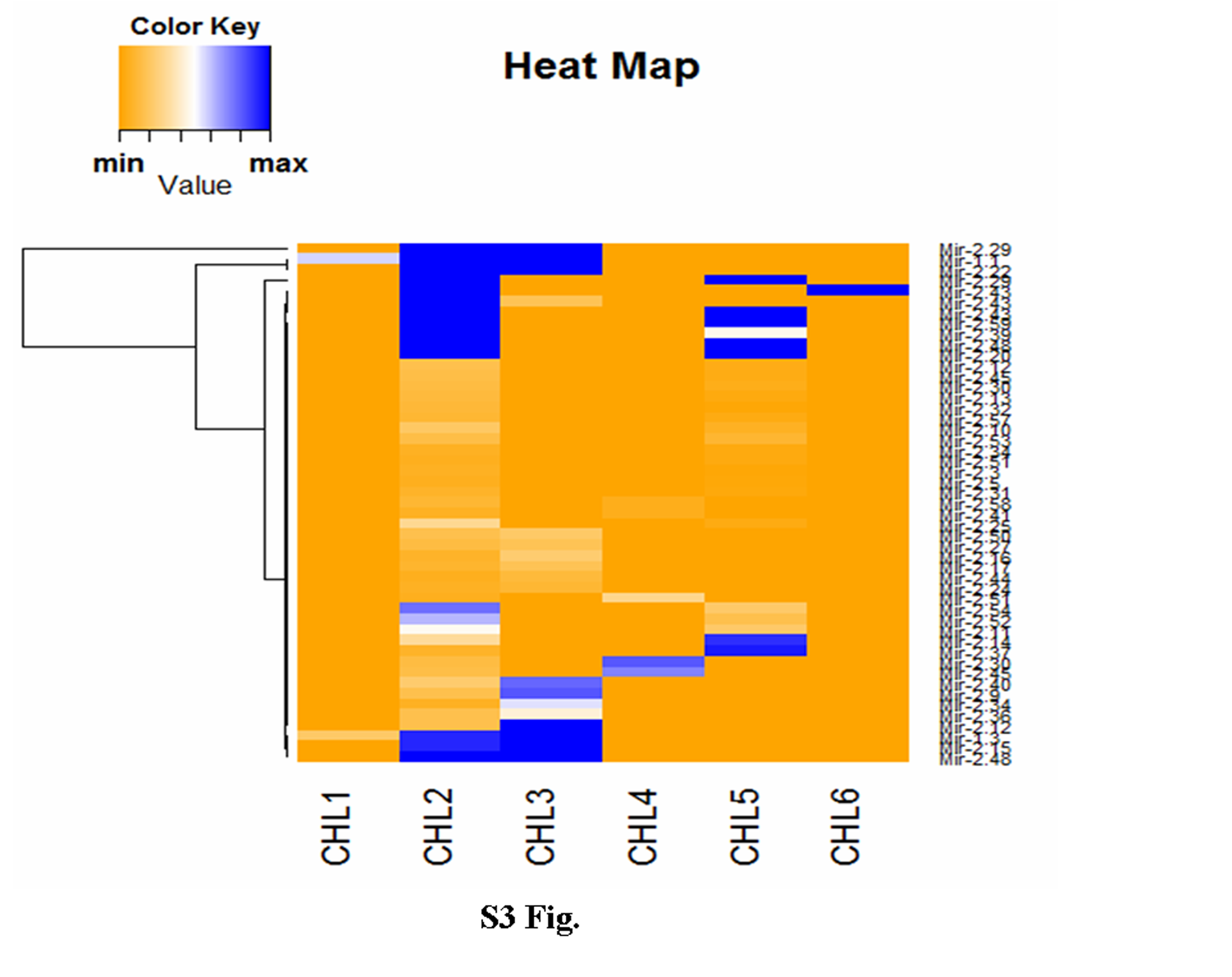

Supplement: S3 Fig — (TIF) [file pone.0139359.s003.TIF]

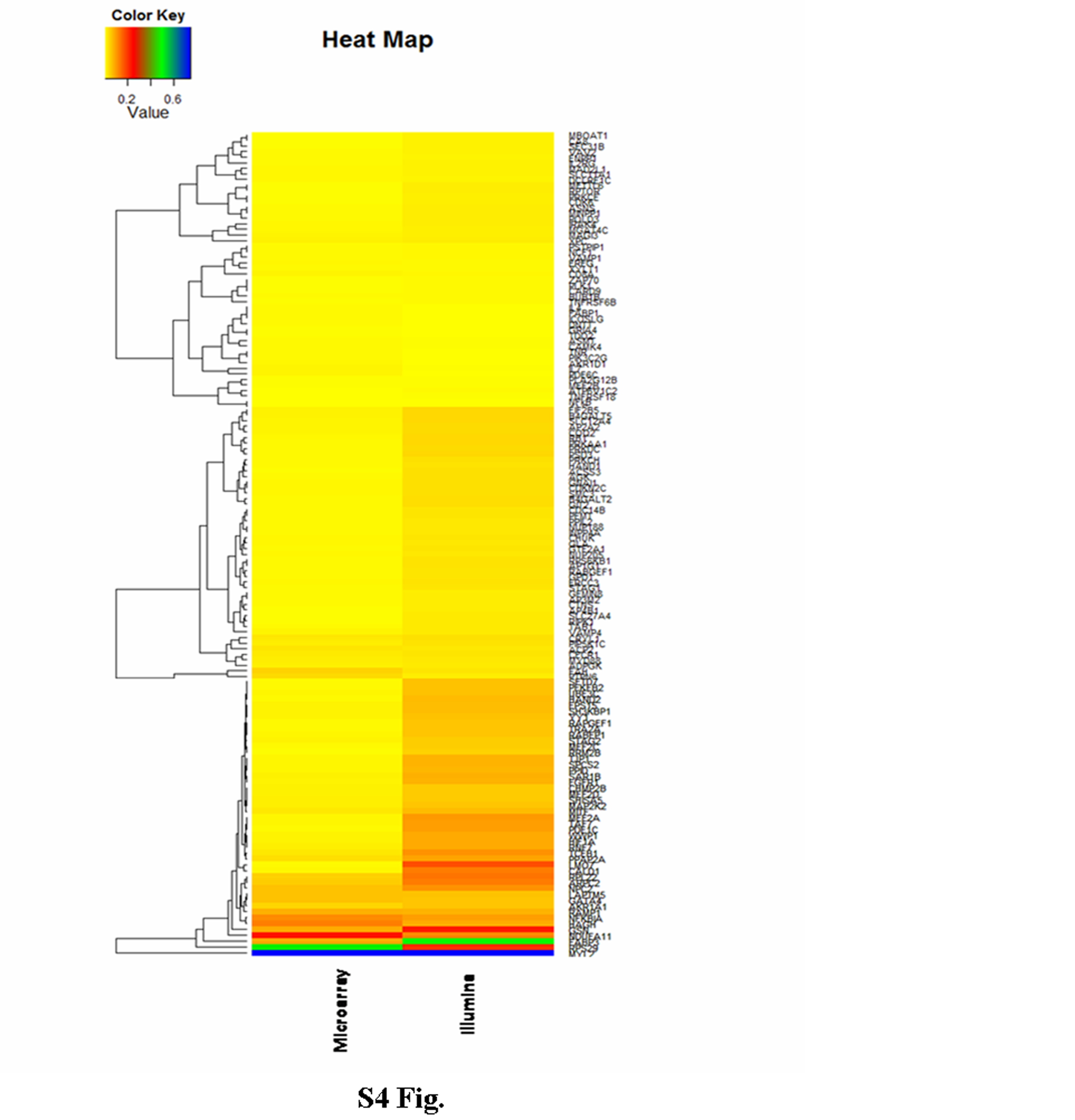

Supplement: S4 Fig — The paired T-test was done to check the significance of expression data using origin 6.1. We found that the obtained data was 95% accurate at 0.05 significant levels. (TIF) [file pone.0139359.s004.TIF]
