# Supplementary material for: Comparative Characterization of Cardiac Development Specific microRNAs: Fetal Regulators for Future
Source: PLoS One. 2015 Oct 14;10(10):e0139359. doi: 10.1371/journal.pone.0139359 (PMC4605649; doi:10.1371/journal.pone.0139359)
Supplement: S1 Table — (DOCX) [file pone.0139359.s005.docx]

| **Sr. No.** | **Conserved MicroRNAs** | **Sequences** |
| --- | --- | --- |
| 1. | Let-7 | Mature miRNA sequence: **5' -**UGAGGUAGUAGGUUGUAUAGU**- 3'**  Probe: 5' - TGGCTCTG - 3'  Forward: 5' - GTGGTGAGGTAGTAGGTTG - 3'  Reverse: 5' - GTGCAGGGTCCGAGGT - 3' |
| 2. | gga-mir-30 | Mature miRNA sequence:**5' -**UGUAAACAUCCCCGACUGGAAG**- 3'**  Probe: 5' - TGGCTCTG- 3'  Forward: 5' - TGTGTTTGTAAACATCCCCGAC - 3'  Reverse: 5' - GTGCAGGGTCCGAGGT - 3' |
| 3. | gga-mir-181 | Mature miRNA sequence: **5' -**AACAUUCAACGCUGUCGGUGAGU**- 3'**  Probe: 5' - ACTCAC- 3'  Forward: 5' - GGAACATTCAACGCTGTCG - 3'  Reverse: 5' - GTGCAGGGTCCGAGGT - 3' |
| 4. | gga-mir-140 | Mature miRNA sequence: **5' –**AGUGGUUUUACCCUAUGGUAG**- 3'**  Probe: 5' - CTACCA- 3'  Forward: 5' - GGGTGGAGTGGTTTTACCCTA - 3'  Reverse: 5' - GTGCAGGGTCCGAGGT - 3' |
| 5. | gga-mir-103 | Mature miRNA sequence:**5'-**UCGGCUUCUUUACAGUGCUGCCUUG **-3'**  Probe: 5' - TGGCTCTG- 3'  Forward: 5' - GGGTTTTCGGCTTCTTTACAGT - 3'  Reverse: 5' - GTGCAGGGTCCGAGGT - 3' |
| 6. | gga-mir-22 | Mature miRNA sequence: **5' -**AGUUCUUCAGUGGCAAGCUUUA**- 3'**  Probe: 5' - CTACCA- 3'  Forward: 5' - GGGAGTTCTTCAGTGGCAA- 3'  Reverse: 5' - GTGCAGGGTCCGAGGT - 3' |
| 7. | Gga-mir-205 | Mature miRNA sequence: **5' –**UCCUUCAUUCCACCGGAGUCUG **- 3'**  Probe: 5' - CTACCA- 3'  Forward: **5' -**GGGTTTCCTTCATTCCACCG**- 3'**  Reverse: 5' - GTGCAGGGTCCGAGGT - 3' |
